# Recombinant BCG expressing the LTAK63 adjuvant increased memory T cells and induced long-lasting protection against *Mycobacterium tuberculosis* challenge in mice

**DOI:** 10.3389/fimmu.2023.1205449

**Published:** 2023-07-13

**Authors:** Lázaro Moreira Marques-Neto, Monalisa Martins Trentini, Alex Issamu Kanno, Dunia Rodriguez, Luciana Cezar de Cerqueira Leite

**Affiliations:** Laboratório de Desenvolvimento de Vacinas, Instituto Butantan, São Paulo, Brazil

**Keywords:** tuberculosis, recombinant BCG, long-term protection, adjuvant, vaccine

## Abstract

Vaccine-induced protection against *Mycobacterium tuberculosis (Mtb)* is usually ascribed to the induction of Th1, Th17, and CD8^+^ T cells. However, protective immune responses should also involve other immune cell subsets, such as memory T cells. We have previously shown improved protection against *Mtb* challenge using the rBCG-LTAK63 vaccine (a recombinant BCG strain expressing the LTAK63 adjuvant, a genetically detoxified derivative of the A subunit from *E. coli* heat-labile toxin). Here we show that mice immunized with rBCG-LTAK63 exhibit a long-term (at least until 6 months) polyfunctional Th1/Th17 response in the draining lymph nodes and in the lungs. This response was accompanied by the increased presence of a diverse set of memory T cells, including central memory, effector memory and tissue-resident memory T cells. After the challenge, the T cell phenotype in the lymph nodes and lungs were characterized by a decrease in central memory T cells, and an increase in effector memory T cells and effector T cells. More importantly, when challenged 6 months after the immunization, this group demonstrated increased protection in comparison to BCG. In conclusion, this work provides experimental evidence in mice that the rBCG-LTAK63 vaccine induces a persistent increase in memory and effector T cell numbers until at least 6 months after immunization, which correlates with increased protection against *Mtb*. This improved immune response may contribute to enhance the long-term protection.

## 1 Introduction

Tuberculosis (TB) is one of the deadliest infectious diseases in the world, responsible for more than 1.3 million deaths in 2021 (1). BCG is the only licensed vaccine against TB, providing protection against severe forms of TB, especially in children. However, as protection wanes, young individuals and adults exhibit variable protection and are more susceptible to pulmonary tuberculosis (2). Given BCG’s excellent safety record, adjuvant properties (heterologous protection), and effectiveness in newborns, several vaccines in development against TB seek to improve BCG’s protection (3–6). In this sense, the vaccine should confer durable protection and induce a prompt and robust immune response against the bacteria in the lungs (the primary site of infection). Therefore, the generation of memory subsets is one of the main goals sought to improve TB vaccines (7, 8).

Classically, the Th1 cells (specially IFN-γ^+^ or polyfunctional cells producing IFN-γ, IL-2 and/or TNF-α) have been considered the most important correlates of protection for TB vaccines. As vaccine development progressed in the field, Th17 and CD8^+^ T cells were also considered important cell populations to induce protective responses (9). In mice, immunization with BCG preferentially induces effector T cells and effector memory T cells (TEM - CD4^+^CD44^+^CD62^-^) and not central memory T cells (TCM - CD4^+^CD44^+^CD62^+^). The effector T cells have an immediate effect but are believed to be vulnerable to exhaustion from chronic infection and continuous exposure to mycobacteria, contrary to TCMs. Another recombinant BCG vaccine, VPM1002 (BCGΔ*ureC*::*hly*) which is in phase III clinical trials, demonstrates that part of its protection against TB is related to an enhancement of the TCM population (10).

Beyond TCM and TEM, tissue-resident memory T cells (TRM - KLRG1^-^PD-1^+^) have also been described as cell subsets involved in protection against TB. KLRG1 and PD-1 are considered important prognosis biomarkers (8, 11). TRM is a memory T cell subset that has a long lifespan in non-lymphoid tissues; they have low body recirculation capacity, but rapidly migrate through the resident organ parenchyma and differentiate into effector cells upon stimulation. In tuberculosis, the pulmonary TRMs were shown to quickly migrate into the lung after adoptive transfer and protect against *Mtb* infection (12–15). The development of TRM, however, was only achieved when BCG was used through the mucosal route, with intradermal/subcutaneous immunization failing to induce this cell population (8, 10). Finally, the level of T cell differentiation (reduced expression of KLRG1 marker, as well as the presence of the inhibitor marker PD-1) can indicate increased IL-2 producer cells that help to maintain effector T cell populations, as well as being less sensitive to exhaustion and apoptosis in chronic infection (12, 16, 17).

The LTK63 is a genetically detoxified *E. coli* heat-labile enterotoxin mutant that exhibits a potent mucosal adjuvanticity. It has been shown that LTK63 can activate several components of the immune response, including the recruitment and activation of neutrophils, NK cells, macrophages, dendritic cells, and B and T cells (18). We have previously developed a recombinant BCG (rBCG) strain expressing the subunit A of LTK63 as an adjuvant (named rBCG-LTAK63). Immunization of mice with rBCG-LTAK63 increased innate and adaptive immune responses and improved the protection against *Mtb* challenge in comparison to BCG (19, 20). Here, we show that the immunization of mice with rBCG-LTAK63 enhances the generation of polyfunctional T cells, TCM, and TEM cells. Six months after immunization, these cells are still in higher numbers. At this time point, mice immunized with rBCG-LTAK63 when challenged with *Mtb*, displayed increased protection as compared with BCG.

## 2 Materials and methods

### 2.1 Animals and immunization

Specific-pathogen-free female BALB/c mice (4–8 weeks old), from Instituto Butantan – Central Animal Facility, were maintained in ABSL-2 racks fitted with a HEPA-filtered air intake and exhaust system. They were kept at the animal care facility of the Laboratório de Desenvolvimento de Vacinas, with water and food provided *ad libitum*. The temperature was maintained from 20–24°C, relative humidity of 40–70%, and a 12 h light/dark cycle. This study was carried out in strict accordance with the Guide for the Care and Use of Laboratory Animals of the Committee of SBCAL (Sociedade Brasileira de Ciência em Animais de Laboratório) recommendations and was approved by the Animal Research Ethical Committee of Instituto Butantan (number: 3435250619).

The rBCG-LTAK63 strain used in this work was previously described (20). BCG or rBCG-LTAK63 were grown in Middlebrook 7H9 (Difco, Detroit, MI, USA) supplemented with 10% of OADC (oleic acid-albumin-dextrose-catalase; BBL, Cockeysville, MD, USA), 0.5% glycerol and 0.05% Tween 80 (7H9-OADC) or plated on Middlebrook 7H10 agar supplemented with 0.5% glycerol and OADC (7H10-OADC).

To evaluate long-term immune response and protection, groups of mice (n=5) were immunized with BCG or rBCG-LTAK63 (1x10^6^ CFU/100 μL) resuspended in phosphate-buffered saline (PBS- 137 mM NaCl, 2.7 mM KCl, 8 mM Na_2_HPO_4_, and 2 mM KH_2_PO_4_) and administered subcutaneously in the back of the animals.

### 2.2 Intranasal infection with *Mtb*

The intranasal infection was performed as described by Logan et al. (2008) (21). A frozen vial of *Mycobacterium tuberculosis* H37Rv (kept at -80°C) was thawed, and the inoculum was adjusted to 1.25x10^4^ CFU/mL with PBS. Ninety and 180 days after immunization, the groups of mice were intranasally challenged with the *Mtb* suspension (500 CFU/40 µL in one nostril). To confirm the bacterial load used, a single mouse from each group was euthanized at day 1 post-inoculation, and the lung homogenates were plated on 7H11-OADC agar. To determine protection, thirty days after infection, animals were euthanized, and the anterior and mediastinal right-lung lobes were collected, homogenized, and plated on 7H11-OADC agar. The bacterial load was determined by counting the CFU numbers after 14-21 days of incubation at 37°C.

### 2.3 Cellular immune responses in draining lymph nodes and lungs

Flow cytometry analysis for specific effector T cell and memory T cell were performed as described in previous protocols (22, 23). Briefly, 90 and 180 days after the immunization, axillar draining lymph nodes and lung lobes were collected. Draining lymph nodes were prepared as single-cell suspensions using 70-µm cell strainers (BD Biosciences), and the cells were resuspended in RPMI-1640 medium supplemented with 10% fetal calf serum, 0.15% sodium bicarbonate, 1% L-glutamine and 1% nonessential amino acids.

Lung lobes were digested with DNAse IV (30 µg/mL) and collagenase III (0.7 mg/mL) for 30 min at 37°C. The digested lungs were prepared as single-cell suspensions using 70-µm cell strainers and erythrocytes lysed using an RBC lysing solution (0.15 M NH_4_Cl, 10 mM KHCO_3_). For both organs, viable cells were counted in a Neubauer chamber using Tripan Blue (0.2%), and cell concentration was adjusted to 1×10^6^ cells/mL. All reagents were purchased from Sigma-Aldrich^®^, Merck KGaA, St. Louis, MO, USA.

Cells were plated in 96-well plates (CellWells™) and stimulated with 10 µg of BCG CFP (*“culture filtrate protein”*, a proteinaceous supernatant of a BCG grown in Sauton medium for 14 days and concentrated through a 5,000 MWCO filter), ConA (positive control) or left unstimulated and incubated at 37°C and 5% CO_2_ for 4 h. Then, monensin (3 µM; eBioscience) was added and cultures were further incubated for another 4 h. Cells were then treated with 0.1% sodium azide (Sigma-Aldrich) in PBS for 30 min at room temperature and centrifuged at 400 x g for 15 min. The cellular phenotype was determined by permeabilization with Perm Fix/Perm Wash (BD Pharmingen) and incubation for 30 min with the following conjugated antibodies: TNF-α-FITC (clone MP6-XT22), IFN-γ-PE (clone XMG1.2), CD4-PerCP (clone RM4-5), CD44-APCcy7 (clone IM7), IL-17-BV421 (clone TC11-18H10), CD62L-FITC (clone MEL-14), PD-1-PE (clone J43), KLRG1-APC (clone 2F1).

Cell acquisition of 70,000 (draining lymph nodes) and 200,000 (lungs) total events per sample was performed using a BD FACS Canto II flow cytometer and data analyzed using FlowJo™ v10 Software (BD Life Sciences).

The CD4^+^ effector T cell population was characterized as to expression of IFN-γ, TNF-α and/or IL-17, either as single, double, or triple-positive cells. Memory T cell population was characterized as: naïve (CD4^+^CD44^-^CD62L^+^), central memory (TCM-CD4^+^CD44^+^CD62L^+^), effector memory (TEM-CD4^+^CD44^+^CD62L^-^), and tissue resident memory (TRM-CD4^+^PD-1^+^KLRG1^-^) cells.

**Figure 1:**
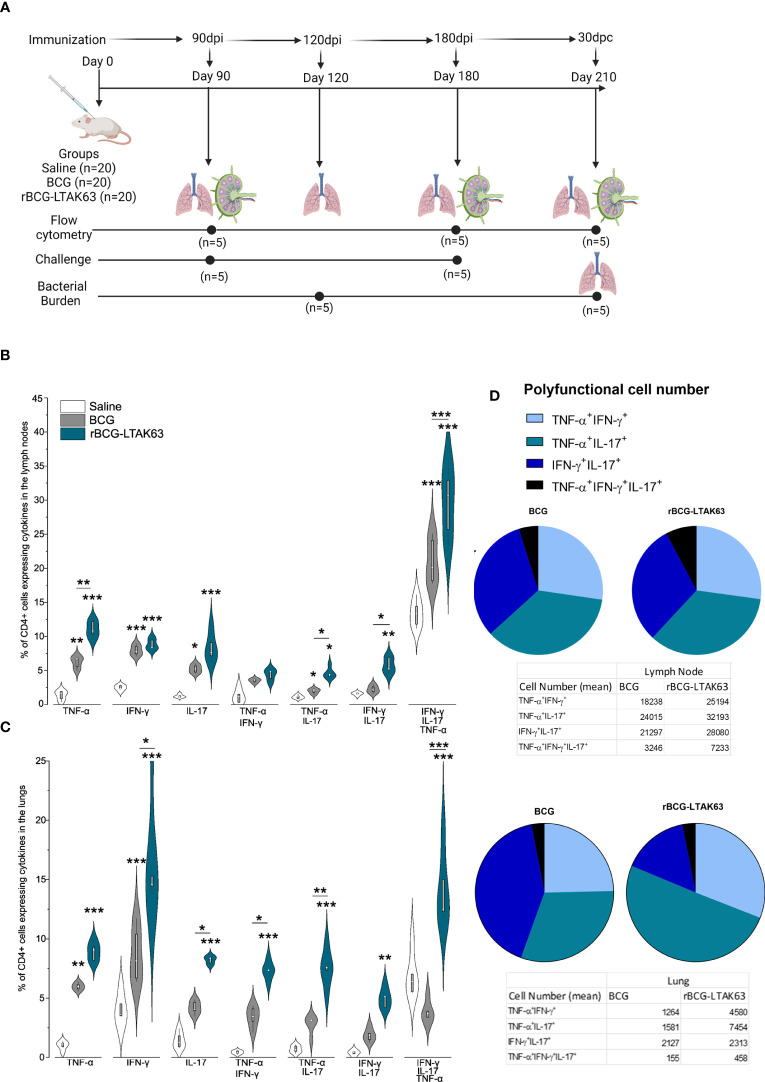
Increased induction of Th1, Th17, and polyfunctional cells in the draining lymph nodes and lungs of rBCG-LTAK63-immunized mice, 90 days after immunization. **(A)** Experimental design of the long-term immune response and protection performed. Created with BioRender.com. Twenty animals were immunized on day 0 with wild-type BCG or rBCG-LTAK63 or mock saline (n=20 per group). Immune responses were evaluated 90 and 180 days after immunization (n=5 per group). Challenges were performed 90 and 180 days after immunization and the protection was evaluated 30 days later (n=5 per group). In the last challenge evaluation (210 days after immunization) the immune response was also measured. Groups of BALB/c mice (n=5/group) were subcutaneously immunized with wild-type BCG or rBCG-LTAK63, and control groups received saline. Axillary lymph nodes **(B)** and lungs **(C)** were collected at 90 days after immunization and cellular suspensions were re-stimulated with CFP (culture filtrate proteins) to evaluate the presence of CD4^+^ single and polyfunctional effector T cell subsets. Violin plots with box whiskers represent the data distribution, median and outliers. **(D)** The pie charts depict the number of polyfunctional cells in evaluated organs. (*) Represents the statistical comparison between groups (*p ≤ 0.05, **p ≤ 0.01, ***p ≤ 0.001). Differences were considered statistically significant when p ≤ 0.05 as compared to saline or BCG group (one-way ANOVA). The (*) above violin plots indicated comparison with the saline control and the (*) bar showed all other group comparisons. The figure shows a representative of two independent experiments.

**Figure 2:**
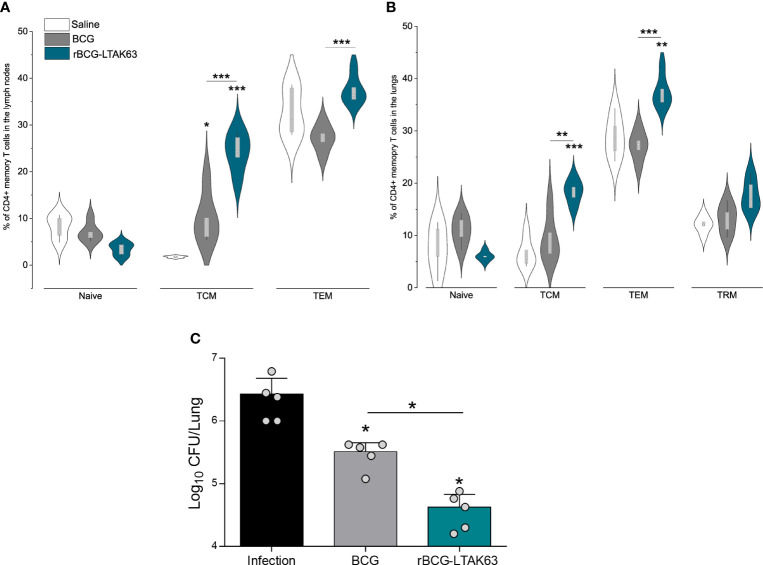
Generation of memory T cells and protection of mice immunized with rBCG-LTAK63, 90 days after immunization. BALB/c mice (n=5/group) were immunized with either BCG or rBCG-LTAK63 (10^6^ CFU); control groups received saline. Lymph node and lung cells were isolated after 90 days and were *in vitro* re-stimulated with CFP to evaluate memory T cell subsets. Memory T cells were characterized as naïve T cells (CD4^+^CD44^-^CD62L^+^), central memory T cells (TCM-CD4^+^CD44^+^CD62L^+^), effector memory T cells (TEM - CD4^+^CD44^+^CD62L^-^) present in the lymph nodes **(A)** and lungs **(B)** of immunized animals. Tissue-resident memory T cells were characterized as CD4^+^PD-1^+^KLRG-1^-^ in the animal’s lungs **(B)**. Violin plots with box whiskers represent the data distribution, median, and outliers. **(C)** Immunized and control animals were challenged intranasally with 500 CFU of *M. tuberculosis* H37Rv 90 days after immunization, and the lung bacillary load was assessed 30 days after infection. (*) Displays the statistical comparison between groups (*p ≤ 0.05, **p ≤ 0.01, ***p ≤ 0.001). Differences were considered statistically significant when p ≤ 0.05 as compared to the saline or BCG group (one-way ANOVA). Bars represent mean ± S.D. The (*) above violin plots indicated comparisons with the saline control and the (*) bar showed all other group comparisons. The figure shows a representative of two independent experiments.

The gating strategy for all memory T cell subsets is shown in **Supplementary Materials**. **Supplementary Figure 1** depicts gating for naïve/TEM/TCM (**Supplementary Figure 1A**) and an example of analysis in the lymph node for each group in both time points (**Figure 1B**). **Supplementary Figure 2** shows gating for TRM (**Supplementary Figure 2A**) and an example of analysis in the lungs for each group in both time points (**Figure 2B**). **Supplementary Figure 3** displays an example of lymph nodes analysis, based on FMO of a single functional T cell, producing IFN-γ (**Supplementary Figure 3A**), TNF-α (**Supplementary Figure 3B**), or IL-17 (**Supplementary Figure 3C**). Cytokine events were background corrected based on this FMO.

The number of cells in each organ was quantified by multiplying the percentage of cells in each gate by the number of live cells counted in the Neubauer chamber.

### 2.4 Statistical analysis

Results were tabulated using the software GraphPad Prism 9 (GraphPad, La Jolla, CA, USA). The violin plot was plotted in Origin (Pro), Version Number (2022b – OriginLab Corporation, Northampton, MA, USA). The differences between groups were assessed using one-way ANOVA. Differences in *p* values < 0.05 were considered statistically significant. All biological experiments were performed at least twice, repeating the immunization and assessments of immune response and protection”.

## 3 Results

### 3.1 rBCG-LTAK63 improves Th1, Th17, memory T cells, and protection, 90 days after immunization

Protection against TB is correlated with an increased Th1/Th17 cytokine response observed at the time of challenge (**Figure 1A**). In agreement, here we show that mice immunized with rBCG-LTAK63 displayed a general increase in the Th1 and Th17 cell populations. At 90 days after immunization, there was an increase of a diverse milieu of CD4^+^ T cells expressing TNF-α, IFN-γ and IL-17 either alone or in combination (double and triple polyfunctional cells) in draining lymph nodes and lungs (**Figures 1B, C**).

In the lymph nodes, rBCG-LTAK63 immunization induced an increased percentage of CD4^+^ single TNF-α and IL-17-producing cells, and combinations of double TNF-α, IFN-γ, and IL-17-producing cells at 90 days. The most significant differences in terms of percentage and in the difference as compared to BCG were in CD4^+^TNF-α^+^ single positive, CD4^+^IFN-γ^+^IL-17^+^ (double positive), and the triple polyfunctional T cells (**Figure 1B**). In the lungs as the target organ, the single CD4^+^ T cells producing IFN-γ and TNF-α, and the double polyfunctional T cells were also increased as compared to BCG. In this case, the largest differences were seen with the CD4^+^IFN-γ^+^ and the triple positive CD4^+^TNF-α^+^IFN-γ^+^IL-17^+^ T cells (**Figure 1C**).

Regarding the numbers of polyfunctional T cells in the lymph node, the triple positive CD4^+^TNF-α^+^IFN-γ^+^IL-17^+^ T cells showed the largest difference compared to BCG (**Figure 1C**). In the lungs, the double positive CD4^+^TNF-α^+^IL-17^+^ T cells were in larger numbers in the rBCG-LTAK63-immunized animals as compared to the BCG group, with a corresponding decrease in the numbers of CD4^+^IFN-γ^+^IL-17^+^ T cells (**Figure 1D**).

Since an increased presence of effector CD4^+^ T cells was observed until 90 days after the immunization with rBCG-LTAK63, we assessed vaccine-induced memory T cells in the draining lymph nodes and lungs of immunized mice. In the lymph nodes, mice immunized with rBCG-LTAK63 displayed a tendency to decrease the naïve T cell population and significantly increased TCM and TEM cells as compared to BCG (**Figure 2A**). In the lungs, the same tendency was observed; in this case, rBCG-LTAK63 immunization displayed significantly larger percentages of the TCM and TEM cell populations. There was a trend to an increase in TRM in rBCG-LTAK63-immunized animals as compared to the saline group; however, this increase was not significant (p value 0.17) (**Figure 2B**).

We had previously shown that rBCG-LTAK63-immunization induces protection against *Mtb* challenge in the intratracheal model of infection, 90 days after immunization (20). Hence, we here confirmed protection against *Mtb* challenge using the intranasal model, 90 days after immunization with rBCG-LTAK63. Animals were administered 500 CFU of *M. tuberculosis* H37Rv intranasally and the bacterial load in the lungs was measured thirty days after the challenge. Also in the intranasal infection model, rBCG-LTAK63 immunization induces better protection than BCG, reducing the bacillary load by more than two logs as compared to the non-immunized group and one log as compared to BCG (**Figure 2C**).

### 3.2 The protective immune response induced by rBCG-LTAK63 immunization is maintained for up to 180 days after immunization

**Figure 3:**
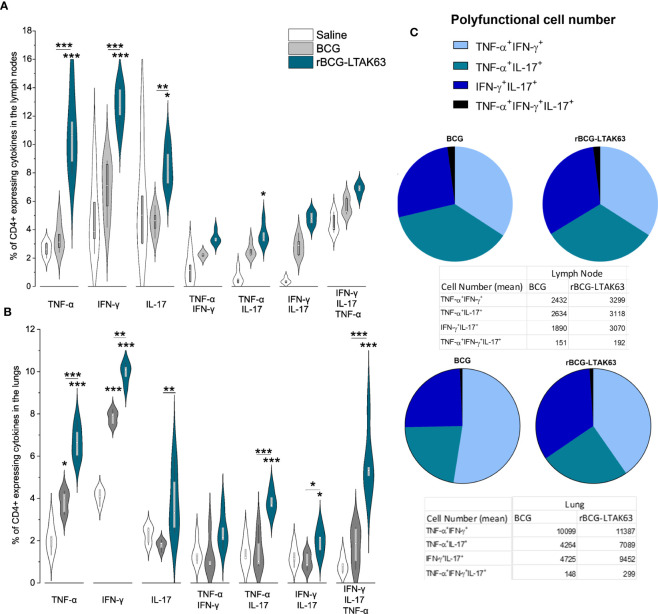
Increased induction of Th1, Th17, and polyfunctional cells in the draining lymph nodes and lungs of rBCG-LTAK63-immunized mice, 180 days after immunization. Groups of BALB/c mice (n=5/group) were subcutaneously immunized with BCG or rBCG-LTAK63; the control group received saline. Axillary lymph nodes **(A)** and lungs **(B)** were collected at 180 days after immunization and cellular suspensions were re-stimulated with CFP (culture filtrate proteins) to evaluate the presence of CD4^+^ effector T cell subsets. Violin plots with box whiskers represent the data distribution, median, and outliers. **(C)** The pie chart depicts the number of polyfunctional cells in evaluated organs. (*) Displays the statistical comparison between groups (*p ≤ 0.05, **p ≤ 0.01, ***p ≤ 0.001). Differences were considered statistically significant when p ≤ 0.05 as compared to saline or BCG group (one-way ANOVA). The (*) above violin plots indicated comparison with the saline control and the (*) bar showed all other group comparisons. The figure shows a representative of two independent experiments.

To determine if the enhanced TEM and TCM cells at 90 days could increase the duration of protection, mice were immunized subcutaneously with 10^6^ CFU (BCG or rBCG-LTAK63), and we assessed TEM and TCM generation, and protection against challenge 180 days later. At 180 days, the CD4^+^ T cells expressing TNF-α, IFN-γ, or IL-17 remained at higher levels in rBCG-LTAK63-immunized animals in comparison to the BCG group in both organs (**Figures 3A, B**). In the lymph nodes, only CD4^+^TNF-α^+^IL-17^+^ double positive is present at a higher level (**Figure 3A**), while in the lungs, CD4^+^TNF-α^+^IL-17^+^, CD4^+^IFN-γ^+^IL-17^+^ double positives are increased, together with the triple-positive T cells (**Figure 3B**).

Regarding the number of polyfunctional T cells in the lymph node, the double positive CD4^+^IFN-γ^+^IL-17^+^ T cells displayed the largest difference compared to BCG (**Figure 3C**). In the lungs, the double positive CD4^+^TNF-α^+^IFN-γ^+^ T cells were in larger numbers in both groups, but the CD4^+^IFN-γ^+^IL-17^+^ T cells were higher in the rBCG-LTAK63 group (**Figure 3C**).

**Figure 4:**
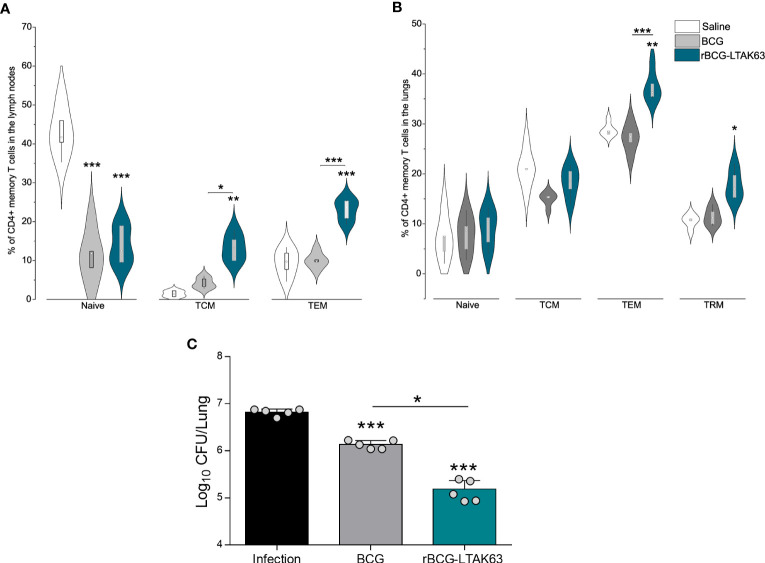
Generation of memory T cells and protection of mice immunized with rBCG-LTAK63, 180 days after immunization. BALB/c mice (n=5/group) were immunized with either BCG or rBCG-LTAK63 (10^6^ CFU); the control group received saline. Lymph node and lung cells were isolated after 180 days and *in vitro* re-stimulated with CFP to evaluate memory T cell subsets. **(A)** Memory T cells were characterized as naïve T cells (CD4^+^CD44^-^CD62L^+^), central memory T cells (TCM-CD4^+^CD44^+^CD62L^+^), effector memory T cells (TEM-CD4^+^CD44^+^CD62L^-^) present in the lymph nodes **(A)** and lungs **(B)** of immunized animals. Tissue-resident memory T cells were characterized as CD4^+^PD-1^+^KLRG-1^-^ in the animal’s lungs **(B)**. Violin plots with box whiskers represent the data distribution, median and outliers. **(C)** Animals were challenged intranasally with 500 CFU of *Mycobacterium tuberculosis* H37Rv 180 days after immunization, and the lung bacillary load was assessed 30 days after infection. (*) Displays the statistical comparison between groups (*p ≤ 0.05, **p ≤ 0.01, ***p ≤ 0.001). Differences were considered statistically significant when p ≤ 0.05 as compared to saline or BCG group (one-way ANOVA). Bars represent mean ± S.D. The (*) above violin plots indicated comparison with the saline control and the (*) bar showed all other group comparisons. The figure shows a representative of two independent experiments.

An increase in TEM cell populations occurs in both organs at 180 days after rBCG-LTAK63 immunization, while there is no alteration of TEM in BCG groups (**Figures 4A, B**). Only rBCG-LTAK63 showed a higher percentage of TCM in the draining lymph node, as compared to BCG (**Figure 4A**). There is also an increase in TRM cells in the lungs of rBCG-LTAK63-immunized animals (**Figure 4B**).

In terms of protection, even after 180 days, rBCG-LTAK63 immunization sustained higher protection against intranasal challenge with *Mtb*, reducing the bacillary load in the animals’ lungs by nearly two logs (**Figure 4C**).

### 3.3 Challenge with *Mtb* induces TEM differentiation and Th1/Th17 recall in animals immunized with rBCG-LTAK63

**Figure 5:**
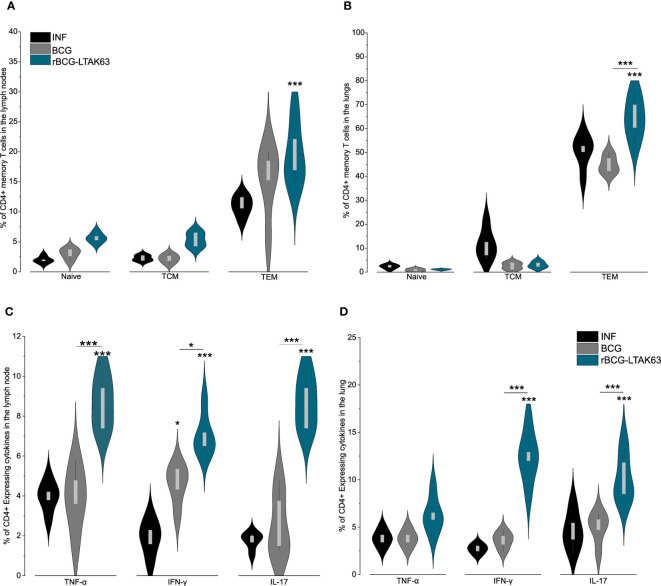
rBCG-LTAK63 induces higher effector and effector memory T cell after infection. BALB/c mice (n=5/group) were immunized with either BCG or rBCG-LTAK63 (10^6^ CFU); the control group received saline. Animals were challenged intranasally with 500 CFU of *Mycobacterium tuberculosis* H37Rv 180 days after immunization; lymph nodes and lung cells were isolated 30 days after infection. Memory T cells were characterized as naïve T cells (CD4^+^CD44^-^CD62L^+^), central memory T cells (TCM-CD4^+^CD44^+^CD62L^+^), effector memory T cells (TEM - CD4^+^CD44^+^CD62L^-^) present in the lymph nodes **(A)** and lungs **(B)** of immunized animals. The lymph nodes **(C)** and lung **(D)** cells were isolated 30 days after infection to evaluate the presence of CD4^+^ effector T cell subsets. Violin plots with box whiskers represent the data distribution, median, and outliers. (*) Displays the statistical comparison between groups (*p ≤ 0.05, ***p ≤ 0.001). Differences were considered statistically significant when p ≤ 0.05 as compared to infection or BCG (one-way ANOVA). The (*) above violin plots indicated comparison with the saline control and the (*) bar showed all other group comparisons. The figure shows a representative of two independent experiments.

Mice were immunized with BCG or rBCG-LTAK63, challenged with *Mtb* 180 days later, and the memory cells (naive, TCM, and TEM) were examined using flow cytometry 30 days later. TEM response in infected animal lymph nodes was higher in animals immunized with rBCG-LTAK63 than in those only infected (**Figure 5A**). In the lungs, TEM was higher in rBCG-LTAK63 group than in both the BCG and infected groups (**Figure 5B**). TCM population showed no significant difference between groups in both organs as compared to BCG.

The increase in the TEM population in the infected animal’s lungs indicates a possible differentiation from TCM into TEM and further into effector cells. Therefore, we also evaluated the Th1 and Th17 responses. The infection with *Mtb* increases CD4^+^TNF-α^+^, CD4^+^IFN-γ^+^, and CD4^+^IL-17^+^ in the lymph nodes of rBCG-LTAK63 immunized animals (**Figure 5C**), while in the lungs, there was a drastic difference in CD4^+^IFN-γ^+^, and CD4^+^IL-17^+^, when compared with BCG (**Figure 5D**).

**Figure 6:**
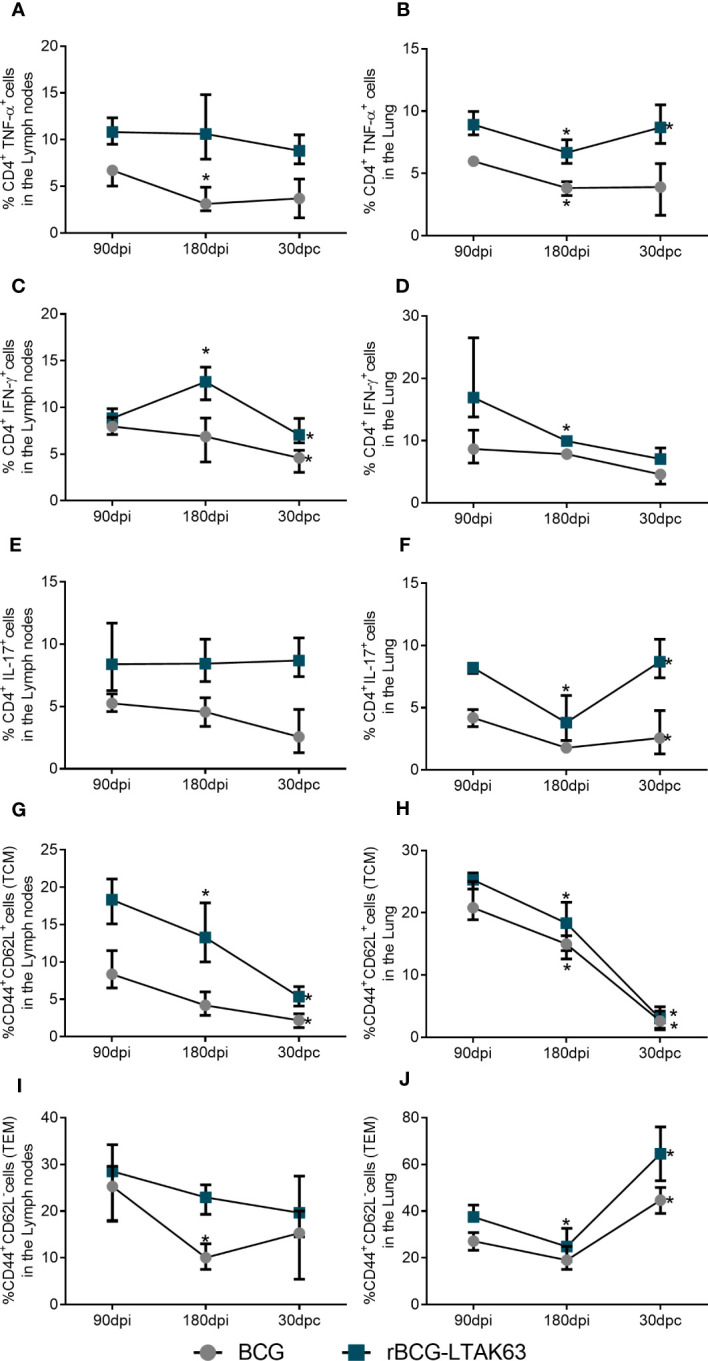
Dynamics of the T cell population show increased TEM and effector T cells after *Mtb* challenge in the lungs of rBCG-LTAK63 immunized animals. Evolution of the cell populations of immunized animals at 90 days (90dpi) and 180 days after immunization (180 dpi), and 30 days after challenge (30dpc): CD4^+^TNF-α^+^ T cells in lymph nodes **(A)** and lungs **(B)**; CD4^+^IFN-γ^+^ T cells in lymph nodes **(C)** and lungs **(D)**; CD4^+^IL-17^+^ T cells in lymph nodes **(E)** and lungs **(F)**; TCM (CD4^+^CD44^+^CD62L^+^) cell populations in lymph nodes **(G)** and lungs **(H)**; TEM (CD4^+^CD44^+^CD62L^-^) in the lymph nodes **(I)** and lungs **(J)**. Bars represent ± S.D. *Statistical difference (p ≤ 0.05) as compared to the prior timepoint in two-way ANOVA test.

Finally, we compared the cell population dynamics across all time points during a longer period of immunization and infection. Regardless of the vaccine used, we can see that after a long period of immunization (180 dpi - before challenge), there is a tendency to decrease in all populations studied, most notably in the lungs of animals (**Figure 6**). Following infection, there is a decrease in the population of TCM cells in both organs and a considerable increase in the TEM cells in the lungs of the animals immunized with rBCG-LTAK63 (**Figures 6G–J**). At the same time, there is an increase in the TNF-α (6B) and IL-17 (6F) producing CD4^+^ T cells in the lungs of the rBCG-LTAK63 group, but they remain stable in the BCG group.

## 4 Discussion

In this study, we show that immunization with rBCG-LTAK63 produces a broader range of effector cells than BCG. It also stimulates the production of more memory cells, primarily TCM. This leads to superior and longer-lasting protection against *Mycobacterium tuberculosis*. To obtain protection against TB, several CD4^+^ T cell subsets should be induced by immunization. Initially, Th1 and Th17 are the main effector cells associated with protection (24). Together, pre-existent TCM, after antigen re-exposure or infection, differentiates into TEM and then into Th1 or Th17 cells that migrate and exert their effector functions in infected tissues. A proportion of these T cells subsequently remain in the lung as TRM and constitute an efficient frontline defense in the organ. These also can turn into Th1/Th17 effector cells, or rapidly recruit new effector cells after infection. In a chronic infection like *Mtb*, the longevity of the immune response and its resistance to continuous antigen exposure without exhaustion, is of equal importance. Hence, cells with lower expression of KLRG1 play a central role, because their proliferative potential can maintain the T cells in the tissue as the infection lasts (8, 25).

The protective mechanism(s) of polyfunctional CD4^+^ T cells induced by vaccines or natural infection are still unknown. However, it has been considered that cells that express multiple effector functions may be more effective at controlling *Mtb* infection than cells that produce a single cytokine. We had previously shown that rBCG-LTAK63 elicited an increased protective response (as compared with BCG) when immunized mice were challenged with H37Rv or a highly virulent Beijing strain (intratracheally) at 90 days after immunization (20). Here we confirmed the previous results in an intranasal challenge model and show that when immunized mice were challenged after 180 days, this improved protection is maintained (**Figures 2**, **4**). Immunization with rBCG-LTAK63 increases Th1 and Th17 single and polyfunctional responses in the lymph node and lungs, for up to 180 days, in contrast to BCG. This long-term protective response is directly associated with the production of Th1 responses, which activate macrophages, stimulate phagocytosis, phagosome maturation, nitrogen reactive production, and improve antigen presentation (26). At the same time, Th17 cells mediate antibacterial and pro-inflammatory responses, contributing to the generation of protective immune responses and memory cells, and support Th1 cell reactivity by down-regulating IL-10 and up-regulating IL-12 production. These responses can protect against tuberculosis infection in the absence of a Th1 response (27, 28).

We have previously demonstrated that intraperitoneal inoculation of rBCG-LTAK63 induced increased recruitment of CD4^+^ lymphocytes (19). Moreover, *in vitro* studies with human macrophages demonstrated that rBCG-LTAK63 upregulated interferon-inducible, antimicrobial, and inflammatory cytokines, and induced tissue repair genes when compared to BCG. Specifically, rBCG-LTAK63-infected macrophages produced higher levels of inflammatory cytokines including IL-12(p70), TNF-α, and IL-15 (29). Our work demonstrates that immunization with rBCG-LTAK63 induces TCM cells in the lymphoid organ (**Figure 2**), as well as TRM cells in the lungs (**Figure 4**). IL-15 (together with IL-7 and IL-2) plays a crucial function in memory T cell development and homeostasis and may explain the TRM and TEM generation. However, the TCM generation seems to be IL-15 independent, and the mechanism by which rBCG-LTAK63 induces TCM is still unknown (30–32). In the TCM and TEM cell population study, it was demonstrated that rBCG-LTAK63 enhances the TCM response and, as expected, this response is maintained in the lymphoid organ while also increased in the animal’s lungs. This improvement is one of the most auspicious characteristics of rBCG-LTAK63 described here. In adoptive transfer studies, TCM generated by VPM1002 immunization was demonstrated to be partly responsible for its increased protection (10).

After the infection, it is expected that the TCM cells differentiate into TEM cells, which migrate from the lymphoid organ to the lungs (3). TCM are not different between the recombinant vaccine and wild-type BCG, while TEM cells are increased in the lungs after infection (**Figures 5**, **6**). This can indicate a possible differentiation of TCM into TEM. Differentiation of TEM will induce an increase in effector T cells (Th1/Th17), and we can see this enhancement in lymph node CD4^+^TNF-α^+^/CD4^+^IFN-γ^+^/CD4^+^IL-17^+^, and in the lungs CD4^+^IFN-γ^+^/CD4^+^IL-17^+^ (**Figure 6**). Our previous work showed that rBCG-LTAK63 reduces NF-κB, IL-12, IFN-γ, TNF-α, and IL-17 after challenge while increasing TGF-β (20). Our results differ from the previous one, most likely due to the method used. In that case, cytokine production was evaluated using RNA transcription, which measures the total cytokine expressed in the tissue. The reduction in total inflammatory cytokine production correlates with the decrease in CFU and in the inflammation area. Here we show the increase in specific T-cell response, which agrees with the later paper that showed an increase in CD4^+^TNF-α^+^ cells in animals immunized with rBCG-LTAK63, fifteen days after H37Rv infection (19).

The long-term protection induced against tuberculosis can be associated with other memory T cells such as the TRM cells; KLRG-1/PD-1 marked T cells are one of the most prominent subsets (16, 17). TRM cells are non-lymphoid tissue memory cells that were shown to be induced in BCG only when the vaccine is intranasally delivered (15, 33). They are considered to be highly protective against tuberculosis (14, 33). Here, the immunization with BCG or rBCG-LTAK63 was performed subcutaneously. Surprisingly, rBCG-LTAK63 improved the generation of TRM (**Figure 4**), which reaches statistical significance at 180 days after immunization. Again, this can be associated to IL-15 production, which also plays an important role in TRM generation and maintenance (30). It is important to observe that a limitation to this study subset is in the characterization of the TRM population. While the expression of PD-1+ KLRG1- has been used as a marker for TRM, these cells can also be found in the vasculature, BAL, and parenchyma. Therefore, in order to confirm that these are actually lung tissue resident cells, we could include CXCR3 as a marker *in vitro* or perform *in vivo* CD45 labeling.

The T CD8 cell populations did not reveal any significant differences between BCG and rBCG-LTAK63 (data not shown). The genetically detoxified LTKA63 protein does not display the same toxicity as LTA, which is an adenylyl cyclase activator; however, LTKA63 maintains part of the adjuvanticity of the original protein. Since neither LTA nor LTAK63 produce cross-presentation, phagosome scape, or any other CD8-inducing function, it was not expected that rBCG-LTAK63 would have this effect. It is important to note that here we do not explore the influence of rBCG-LTAK63 on crucial cell populations involved in tuberculosis protection and protective immunity development (i.e., dendritic cells, monocytes, macrophages), and we use a single gender and mouse strain (34, 35). The next stages should address these limitations using mice strains with diverse tuberculosis susceptibility (e.g., CBA, C3HeB/FeJ, DBA/2, and 129SvJ), different animal genders, and evaluating other possible processes associated with the rBCG-LTAK63 protective effect.

Overall, our findings show that rBCG-LTAK63 immunization increased the levels of several memory T cell subsets, which correlates with the longer-lasting protection observed against challenge. These findings suggest that rBCG-LTAK63 can induce a more durable and stable immune response and protection, which could address some of the current BCG vaccine issues.

## Data availability statement

The raw data supporting the conclusions of this article will be made available by the authors, without undue reservation.

## Ethics statement

The animal study was reviewed and approved by 3435250619.

## Author contributions

LM-N, MT, DR, AK, and LL conceived and designed the experiments; MT and LM-N performed the experiments and collected data; LM-N, MT, DR, AK, and LL processed and analyzed the data; LM-N, MT, AK, and LL wrote the manuscript, and all authors critically revised the manuscript.
