# Supplementary material for: Recombinant BCG expressing the LTAK63 adjuvant increased memory T cells and induced long-lasting protection against Mycobacterium tuberculosis challenge in mice
Source: Front Immunol. 2023 Jul 13;14:1205449. doi: 10.3389/fimmu.2023.1205449 (PMC10374402; doi:10.3389/fimmu.2023.1205449)
Supplement: Supplementary file 1 [file DataSheet_1.docx]

**Recombinant BCG expressing the LTAK63 adjuvant increased memory T cells and induced long-lasting protection against *Mycobacterium tuberculosis* challenge in mice**

**Lázaro Moreira Marques-Neto^1^, Monalisa Martins Trentini^1^, Alex Issamu Kanno^1^, Dunia Rodriguez^1^, Luciana Cezar de Cerqueira Leite^1*^**

Laboratório de Desenvolvimento de Vacinas. Instituto Butantan, Brazil.

*Correspondence:

Luciana Cezar de Cerqueira Leite

e-mail: [luciana.leite@butantan.gov.br](mailto:luciana.leite@butantan.gov.br)

Postal address: Laboratório de Desenvolvimento de Vacinas. Instituto Butantan. São Paulo, Brazil. Avenida Doutor Vital Brasil, 1500. ZIP-CODE: 05.503-900

## Supplementary Figures

##
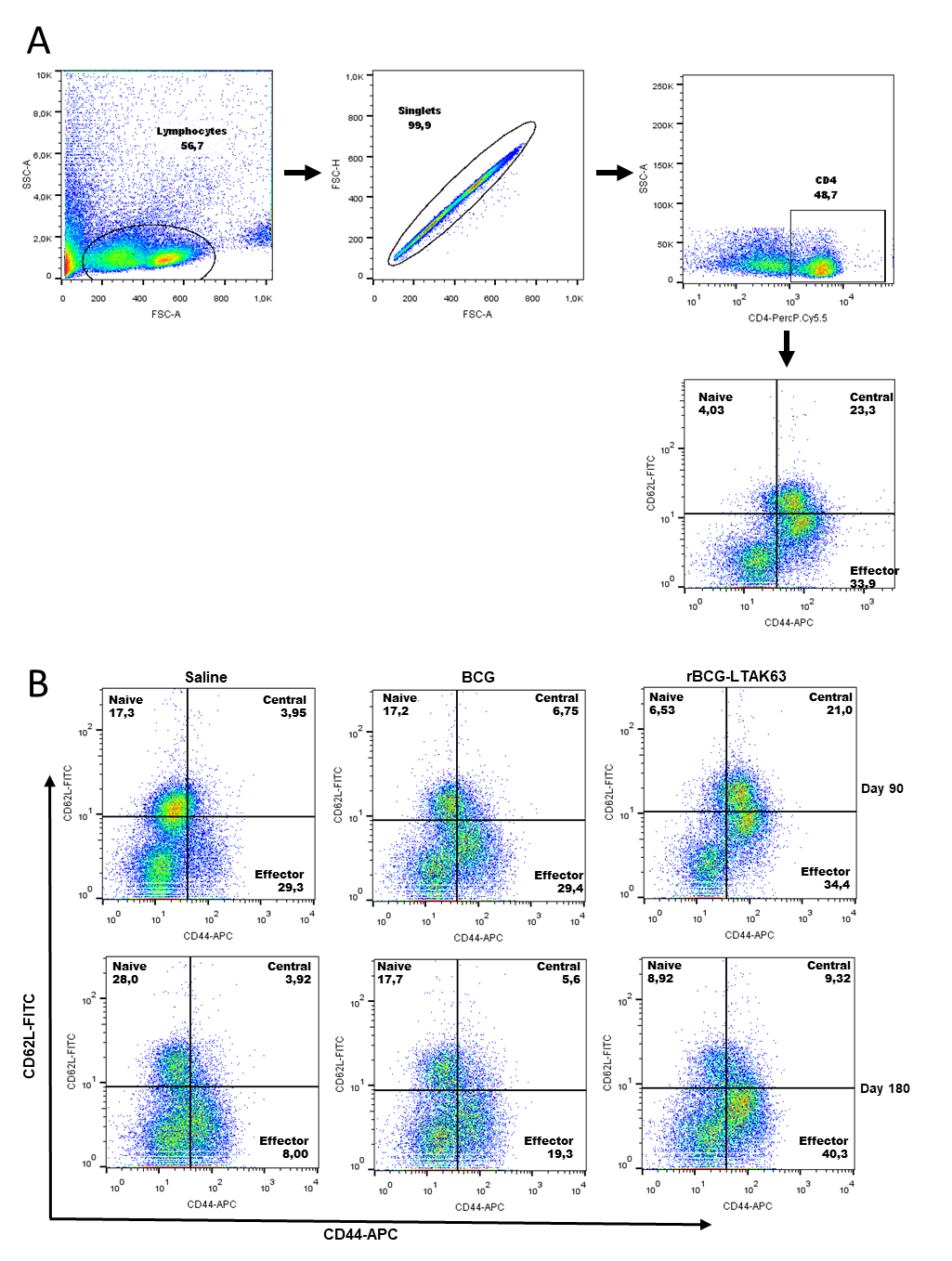


**Supplementary Figure 1.** **Representative FACS plots showing the gating strategy to determine the frequency of naïve T cell, central memory T cell (TCM) and effector memory T cell (TEM)**. (A) Flow cytometry plots from lymph nodes showing the gating scheme analyzing, naive TCM and TEM. (B) Representative flow cytometry plots showing the frequency of naïve/TCM/TEM analysis in the lymph node, for all groups in both time points, before the challenge.

##
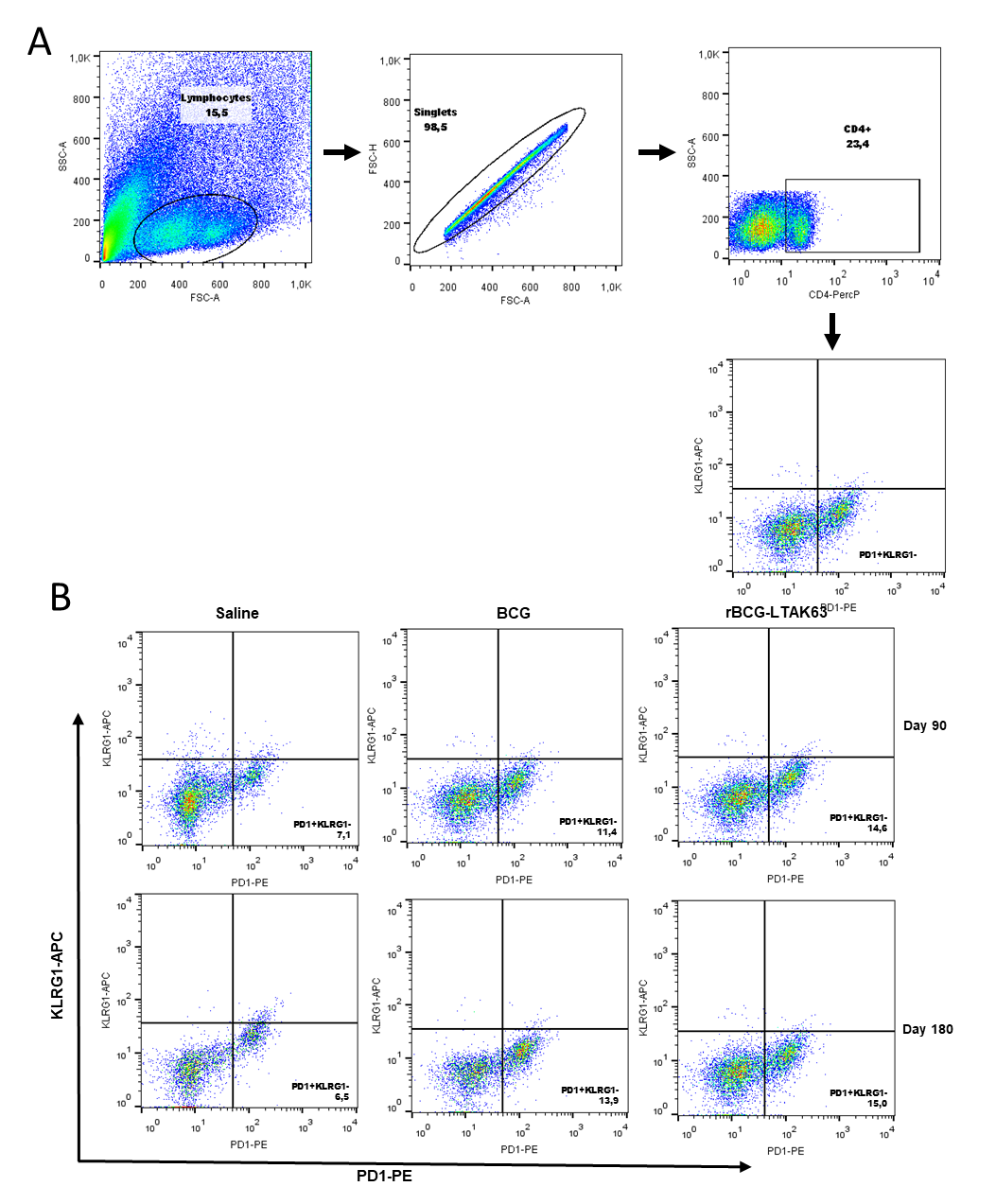


**Supplementary Figure 2.** **Representative FACS plots showing the gating strategy to determine the frequency of tissue-resident memory T cell (TRM).** (A) Flow cytometry plots from lymph nodes showing the gating scheme analyzing, TRM. (B) Representative flow cytometry plots showing the analysis of frequency of TRM in the lungs, for all groups in both time points, before the challenge.


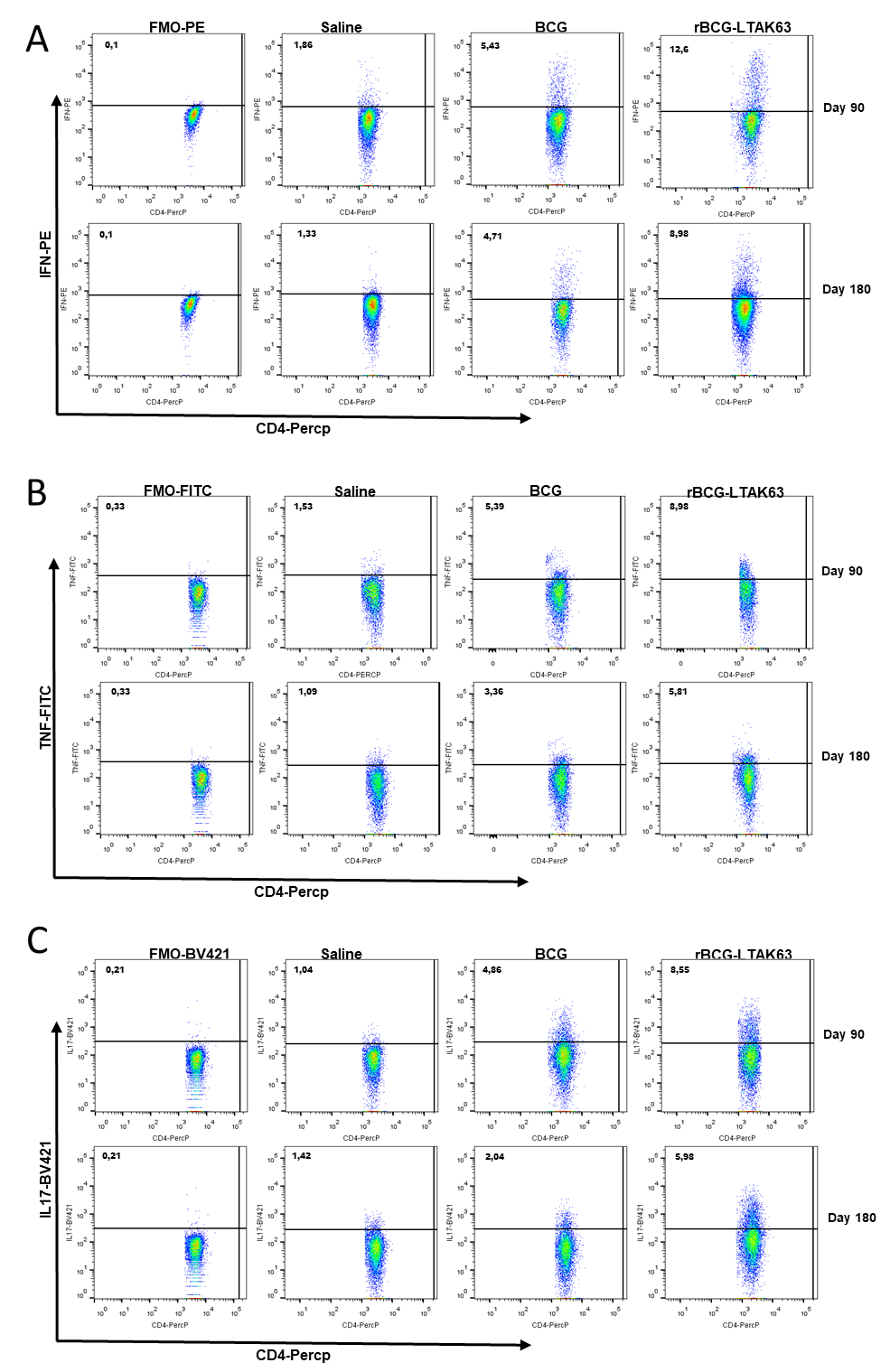


**Supplementary Figure 3.** **Representative FACS plots showing the frequency of single-positive cytokine-producing T cells in the lymph nodes, before challenge**. (A) Flow cytometry plots from lymph nodes showing the frequency of T CD4+ (PercP) and IFNγ+ (PE). (B) Flow cytometry plots from lymph nodes showing the frequency of T CD4+ (PercP) and TNF-α+ (FITC). (C) Flow cytometry plots from lymph nodes showing the frequency of T CD4+ (PercP) and IL-17+ (BV421). All analyses were based on Fluorescence Minus One - FMO (first column).
